# Home care as a safe alternative in post-acute and long-term care during COVID-19 crisis

**DOI:** 10.31744/einstein_journal/2020CE6053

**Published:** 2020-10-20

**Authors:** Heloisa Amaral Gaspar, Cláudio Flauzino de Oliveira, Fabiana Camolesi Jacober

**Affiliations:** 1 Home DoctorSão PauloSPBrazil Home Doctor , São Paulo , SP , Brazil .

Dear Editor,

Advanced age and comorbidities are associated with increased mortality related to coronavirus disease 2019 (COVID-19). The high prevalence of this combination, associated with physical environments that provide inadequate barriers to infection control, place patients in long-term care facilities at great risk. There are several reports worldwide about the high mortality related to COVID-19 among residents of long-term care institutions, showing they account for 25% of deaths from COVID-19, in the United States. ^( 1 , 2 )^ Percentages are even higher in some US states and European countries. ^( 3 )^

In Brazil, much of post-acute care and long-term chronic patient care is provided at home. Our organization provided home care to 2,931 patients in the first 3 months of the pandemic and reported only 31 cases of COVID-19 (1%) and six deaths. The low incidence of COVID-19 in this population reinforces that home care protects patients and lessens the risk of infections. Patients naturally remain at home isolation and are treated by a team of professionals in a directed way. This, together with proper use of personal protective equipment (PPE) and implementation of innovations ( *e.g.* telemedicine), are key for safe care.

Shifting post-acute and chronic care to the household environment with the implementation of technology should be a recommended alternative.
