# Supplementary material for: Home care as a safe alternative in post-acute and long-term care during COVID-19 crisis
Source: Einstein (Sao Paulo). 2020 Oct 20;18:eCE6053. doi: 10.31744/einstein_journal/2020CE6053 (PMC7575037; doi:10.31744/einstein_journal/2020CE6053)
Supplement: Translation

# Translation: *Home care* como alternativa segura no cuidado pós-agudo e crônico durante crise por COVID-19

**Authors:** Heloisa Amaral Gaspar, Cláudio Flauzino de Oliveira, Fabiana Camolesi Jacober

**Affiliations:** 1 Brasil Home Doctor, São Paulo, SP, Brasil .

Caro Editor,

Idade avançada e comorbididades estão associadas à maior mortalidade por doença pelo coronavírus 2019 (COVID-19). A alta prevalência dessa combinação, associada a ambientes físicos que oferecem barreiras inadequadas para controle de infecção, coloca os pacientes de instituições de longa permanência em maior risco. Há vários relatos mundiais sobre a alta mortalidade relacionada à COVID-19 em residentes dessas instituições, chegando a 25% dos óbitos por COVID-19 nos Estados Unidos. ^( 1 , 2 )^ As percentagens são ainda maiores em alguns estados americanos e em países europeus. ^( 3 )^

No Brasil, a maior parte do cuidado pós-agudo e de pacientes crônicos é oferecido em casa. Nossa organização propiciou atendimento domiciliar a 2.931 pacientes nos primeiros 3 meses da pandemia e registrou apenas 31 casos de COVID-19 (1%) e seis mortes. A baixa incidência da COVID-19 nessa população reforça que o atendimento domiciliar protege pacientes e diminui o risco de infecções. Os pacientes permanecem naturalmente em isolamento domiciliar e são tratados por uma equipe assistencial de forma dirigida. Essa abordagem, junto do uso apropriado de Equipamentos de Proteção Individual (EPI) e da implementação de inovações (por exemplo, telemedicina), é essencial para um cuidado seguro.

Levar o cuidado pós-agudo e crônico para o ambiente domiciliar, com implementação de tecnologias, deve ser uma alternativa recomendada.
